# Supplementary material for: Early-life-trauma triggers interferon-β resistance and neurodegeneration in a multiple sclerosis model via downregulated β1-adrenergic signaling
Source: Nat Commun. 2021 Jan 4;12:105. doi: 10.1038/s41467-020-20302-0 (PMC7782805; doi:10.1038/s41467-020-20302-0)
Supplement: Supplementary file 1 — Supplementary Information [file 41467_2020_20302_MOESM1_ESM.pdf]

Early-life-trauma triggers interferon-β resistance and neurodegeneration in a multiple sclerosis model via downregulated β1-adrenergic signaling

Khaw et al.

Corresponding Author:
Makoto Inoue
Illinois at Urbana-Champaign, Urbana, IL, USA
Telephone: +1-217-300-4085
E-mail address: makotoi@illinois.edu

This work was supported by University of Illinois start-up funds (MI). The authors have no financial or personal conflict of interest.

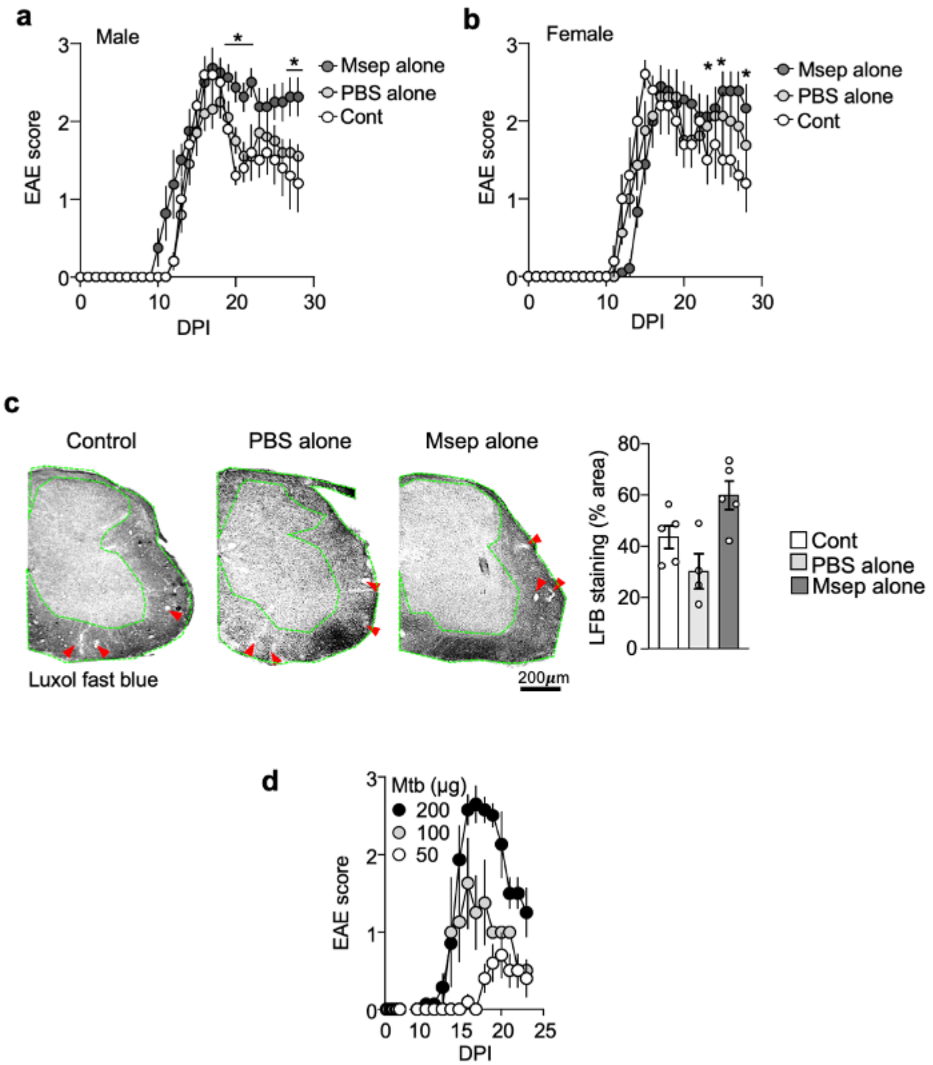

Supplementary Figure 1: Disease severity and demyelination status of control conditions

(a, b) Mean clinical EAE scores and cumulative scores of **(a)** male mice and **(b)** female mice subjected to nothing, maternal separation only (Msep alone), or neonatal PBS injection only (PBS alone) then induced with EAE in adulthood (n=5 animals/group).

(c) Spinal cord ventral root sections of EAE mice stained for myelin using Luxol fast blue at 30 dpi (Control: n=5, PBS alone; n=4. Msep alone; n=5 animals) with quantitative analysis of stained area. Green outline indicates analyzed white matter region of interest. Red arrows indicate demyelination regions.

(d) Titrated Mtb dosage (μg/mouse) using EAE induction to determine optimal dose for weak EAE induction to examine EAE susceptibility (50 μg/mouse; n=5, 100 μg/mouse; n=4, 200 μg/mouse; n=4). Each dot represents averaged data per animal. Data is represented as mean ± SEM. Data is represented as mean ± SEM. Two-tailed student's *t*-test, *$P < 0.05$, compare between control and Msep groups (a, b). Exact p-values for asterisks: **(a)** 0.0178, **(b)** 0.0305.

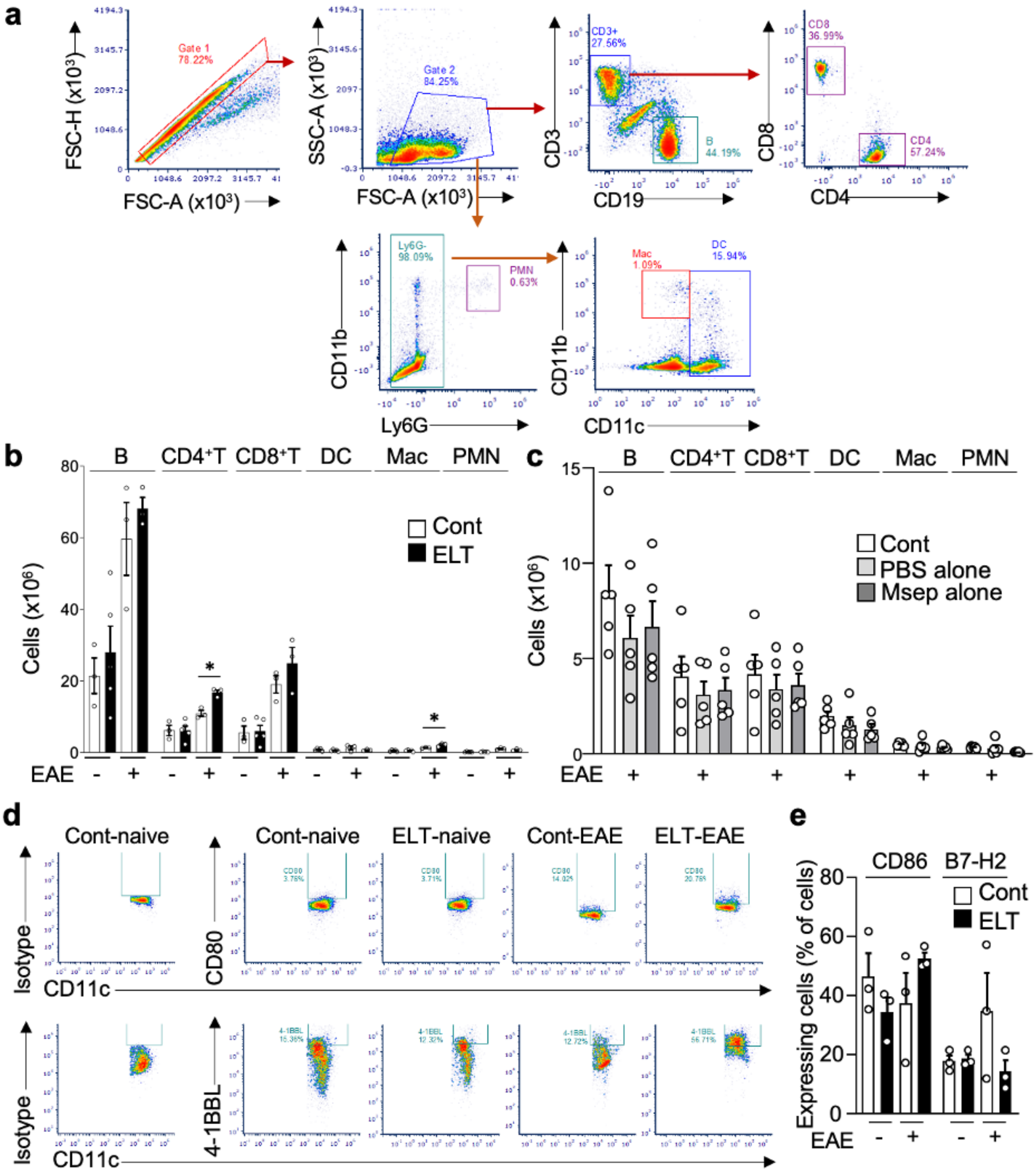
a
FSC-H (x10³)
FSC-A (x10³)
Gate 1
78.22%
SSC-A (x10³)
Gate 2
84.25%
CD3
CD19
CD3+
27.56%
B
44.19%
CD8
CD4
CD8
36.99%
CD4
57.24%
CD11b
Ly6G
Ly6G-
98.09%
PMN
0.63%
CD11c
Mac
1.09%
DC
15.94%
b
B
CD4⁺T
CD8⁺T
DC
Mac
PMN
Cells (x10⁶)
Cont
ELT
EAE
c
Cont
PBS alone
Msep alone
d
Cont-naive
ELT-naive
Cont-EAE
ELT-EAE
Isotype
CD11c
CD80
4-1BBL
e
CD86
B7-H2
Expressing cells (% of cells)

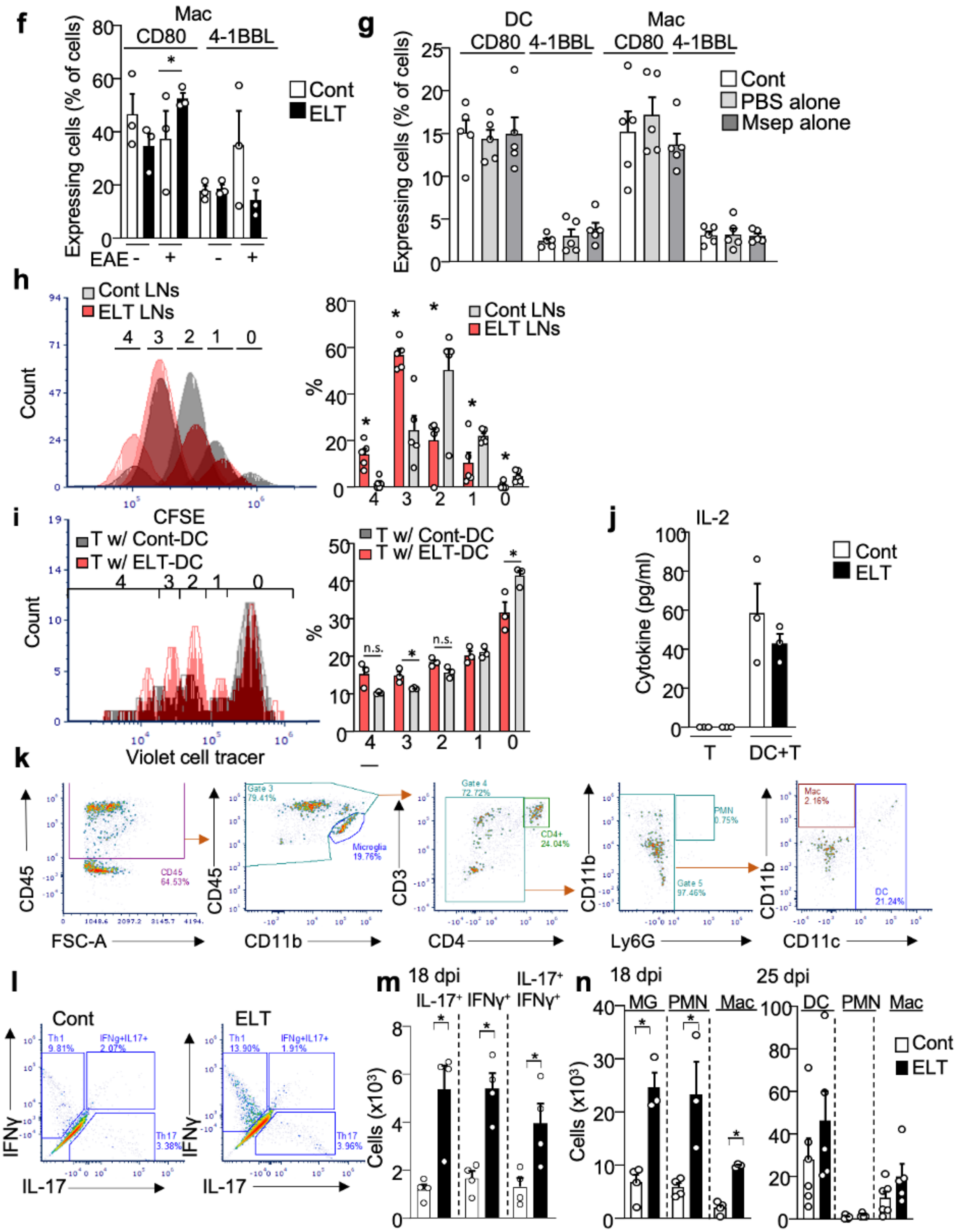
f
Mac
CD80
4-1BBL
Expressing cells (% of cells)
Cont
ELT
EAE - + - +
g
DC
CD80 4-1BBL
Mac
CD80 4-1BBL
Expressing cells (% of cells)
Cont
PBS alone
Msep alone
h
Cont LNs
ELT LNs
4 3 2 1 0
Count
%
i
CFSE
T w/ Cont-DC
T w/ ELT-DC
Count
Violet cell tracer
n.s.
j
IL-2
Cytokine (pg/ml)
Cont
ELT
T
DC+T
k
Gate 3
79.41%
Microglia
19.76%
Gate 4
72.72%
CD4+
24.04%
PMN
0.75%
Gate 5
97.46%
Mac
2.16%
DC
21.24%
CD45
64.53%
CD45
FSC-A
CD11b
CD3
CD4
Ly6G
CD11c
l
Cont
ELT
Th1
9.81%
IFNg+IL17+
2.07%
Th17
3.38%
Th1
13.90%
IFNg+IL17+
1.91%
Th17
3.96%
IFNγ
IL-17
m
18 dpi
IL-17⁺ IFNγ⁺
IL-17⁺ IFNγ⁺
Cells (x10³)
n
18 dpi
MG PMN Mac
25 dpi
DC PMN Mac
Cells (x10³)
Cont
ELT

Supplementary Figure 2: Increased peripheral immune cell activation under ELT-EAE condition.

(a) Representative gating strategy illustrating populations of immune cells, namely B cell: $CD19^+$, $CD4^+$T cell: $CD3^+CD4^+$, $CD8^+$T cell: $CD3^+CD8^+$, dendritic cell: $CD11c^+$, macrophage: $CD11b^+CD11c^-$, and neutrophil: $CD11b^+Ly6G^{high}$.

(b) Splenic immune cell number of control and ELT mice at EAE 10 dpi (Cont-naïve: n=3, ELT naïve: n=5, Cont-EAE : n=3, ELT-EAE: n=3).

(c) Lymph node immune cell number of control, PBS alone, and Msep alone mice at 10 dpi (n=5 animals/group).

(d) Representative gating strategy illustrating dendritic cell ($CD11c^+$) expression of CD80 and 4-1BBL.

(e) Percentage of CD86-, B7-H2-expressing dendritic cell harvested from lymph nodes at 10 dpi (n=5 animals/group).

(f) Percentage of CD80 and 4-1BBL-expressing macrophage harvested from lymph nodes at 10 dpi (n=5 animals/group).

(g) Percentage of CD80 and 4-1BBL expressing dendritic cell or macrophage harvested from lymph nodes at 10 dpi (n=5 animals/group).

(h) Proliferation assay of $CD4^+$T cells ($CD3^+CD4^+$) in lymph node-derived cells isolated from control EAE and ELT EAE mice at 10 dpi (n=3 animals/group).

(i) Proliferation assay of naïve 2D2 T cell 72 hours post co-culture with dendritic cells (DC) isolated from control EAE or ELT-EAE mice at 10 dpi (n=5 animals/group).

(j) Amount of IL-2 cytokine in supernatant derived from culture conditions 72 hours post culture initiation. CD4T cells were isolated from draining lymph nodes of ELT-EAE animals on 10 dpi to be supplemented with MOG (10 ug/ml) and cultured alone or with isolated dendritic cell (n=3 animals/group).

(k) Representative gating strategy illustrating populations of spinal cord infiltrated immune cells, namely microglia: $CD45^{low}$ $CD11b^+$, $CD4^+$T cell: $CD45^{high}CD3^+CD4^+$, Neutrophil: $CD45^{high}CD11b^+Ly6G^{high}$, Dendritic cell: $CD45^{high}CD11c^+$, Macrophage: $CD45^{high}CD11b^+CD11c^-$ at 18 dpi (n=4 per group).

(l) Representative gating strategy illustrating T cell expression of intracellular cytokines: IL-17, interferon γ (IFNγ) to distinguish populations of IL-17^+ Th17 cells, IFNγ^+ Th1 cells, and IL-17^+ IFNγ^+ cells (n=5 animals/group).

(m) Absolute numbers of IL-17^+ secreting $CD4^+$T (Th17), interferon γ^+ secreting $CD4^+$T cells (Th1), and IL-17^+ interferon γ^+ $CD4^+$T cells in the spinal cord of EAE mice at 18 dpi (n=4 animals/group).

(n) Absolute numbers of microglia (MG: $CD45^{low}CD11b^+$), macrophage (Mac: $CD45^{high}CD11b^+Ly6G^-$ $CD11c^-$), and neutrophils (PMN: $CD45^{high}CD11b^+Ly6G^+$) in the spinal cord at 18 dpi (Cont: n=4, ELT: n=3); Absolute numbers of dendritic cell (DC: $CD45^{high}CD11c^+$), macrophage and neutrophils in the spinal cord at 25 dpi (n=5 per group). Each dot represents averaged data per animal. Data is represented as mean ± SEM. Two-tailed student's *t*-test, *$P < 0.05$. Exact p-values for asterisks: (b) 0.0056, 0.036, (f) 0.00086, (h) 0.0159, 0.0452, 0.0211, 0.0017, 0.0016, (i) 0.0416, 0.0426, (m) 0.00661, 0.001737, 0.0265, (n) 0.0021, 0.002, 0.000098.

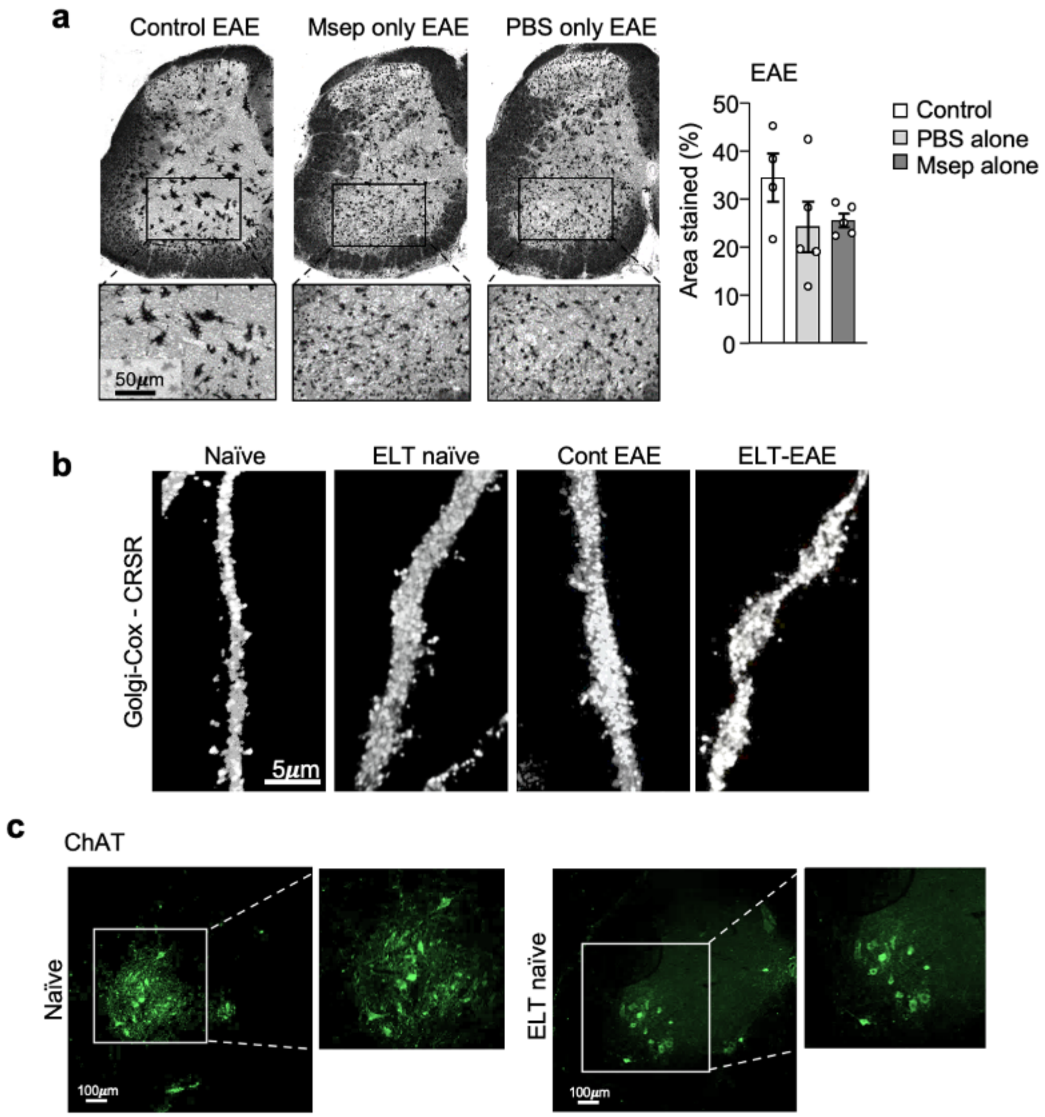

Supplementary Figure 3: Neuron status in motor-related ventral horn of lumbar spinal cord.

(a) Golgi-Cox stained lumbar spinal cord sections of EAE mice isolated at 30 dpi with quantification of area stained/per field (Cont EAE: n=4; PBS alone EAE: n=5; Msep alone EAE: n=5).

(b) Representative images of Golgi-Cox stained dendrite and dendritic spines in the lumbar spinal cord ventral region**.** Representative image was randomly selected from pooled images (n=3 animals/group, 60 dendrites/group).

(c) Representative images of cholinergic motor neurons in the ventral lumbar spinal cord regions of control-naïve and ELT-naive mice. Representative image was randomly selected from pooled images from at least two independent staining experiments.

Each dot represents averaged data per animal. Data is represented as mean ± SEM.

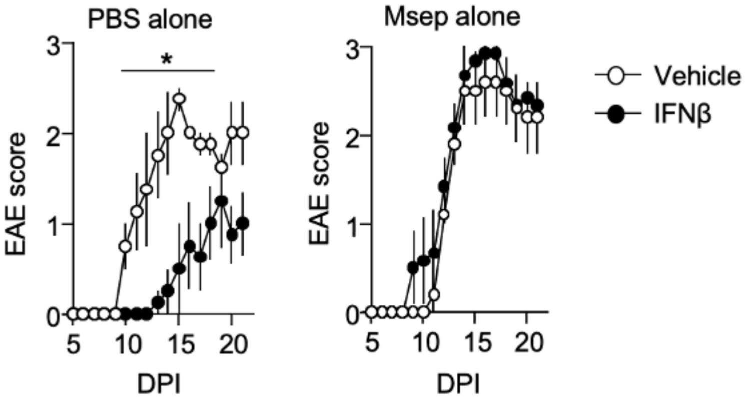

Supplementary Figure 4: Maternal separation alone was sufficient to induce interferon beta resistance.

EAE scores of naïve, Msep alone, and PBS alone IFNβ-treated mice, (PBS alone-vehicle: n=4, PBS alone-IFNβ; n=4, Msep alone-vehicle: n=5, Mspe alone-IFNβ: n=6). IFNβ was treated from 0 dpi to 9 dpi once every 3 days. Data is represented as mean ± SEM. Data is represented as mean ± SEM. Two-tailed student's *t*-test, *$P < 0.05$. Exact p-values for asterisks: 0.010.

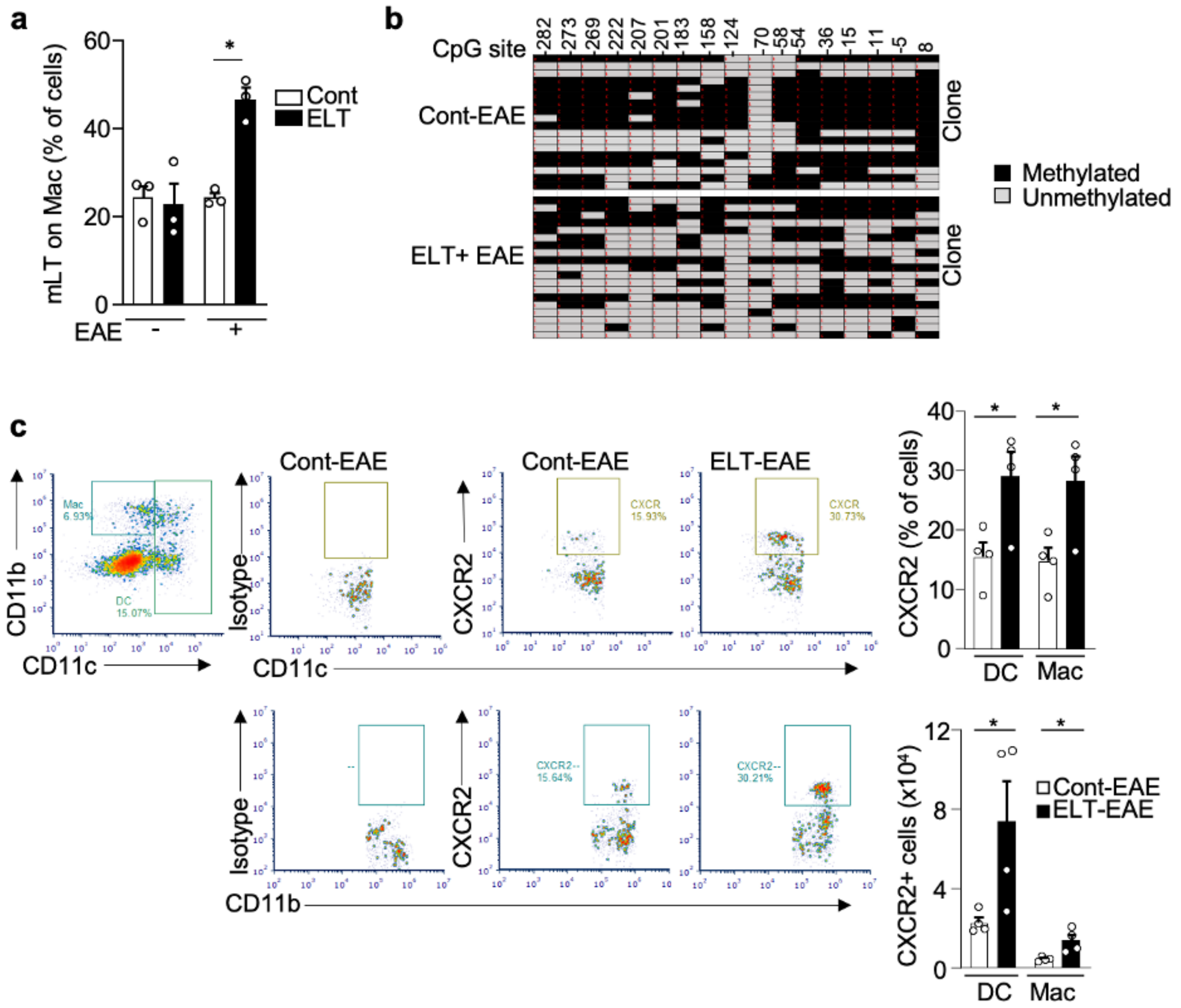

Supplementary Figure 5: ELT changes *Lta* methylation status and CXCR2 expression in DCs.

(a) Percentages of mLT expressing macrophages derived from lymph nodes of control EAE mice and ELT-EAE mice (n=3 animals/group).

(b) Bisulfite sequencing verification of the DNA methylation status of the indicated CpG island regions within the *Lta* promoter gene of isolated DCs (n=3 animals/group).

(c) Representative gating strategy illustrating dendritic cell and macrophage expression of CXCR2. Percentage and absolute numbers of CXCR2 expressing DCs and macrophages derived from lymph nodes. (n=4 animals/group).

Data is represented as mean ± SEM. Student's t-test, *$P < 0.05$. Exact p-values for asterisks: **(a)** 0.0016 **(c)** 0.029, 0.026, 0.049, 0.019.

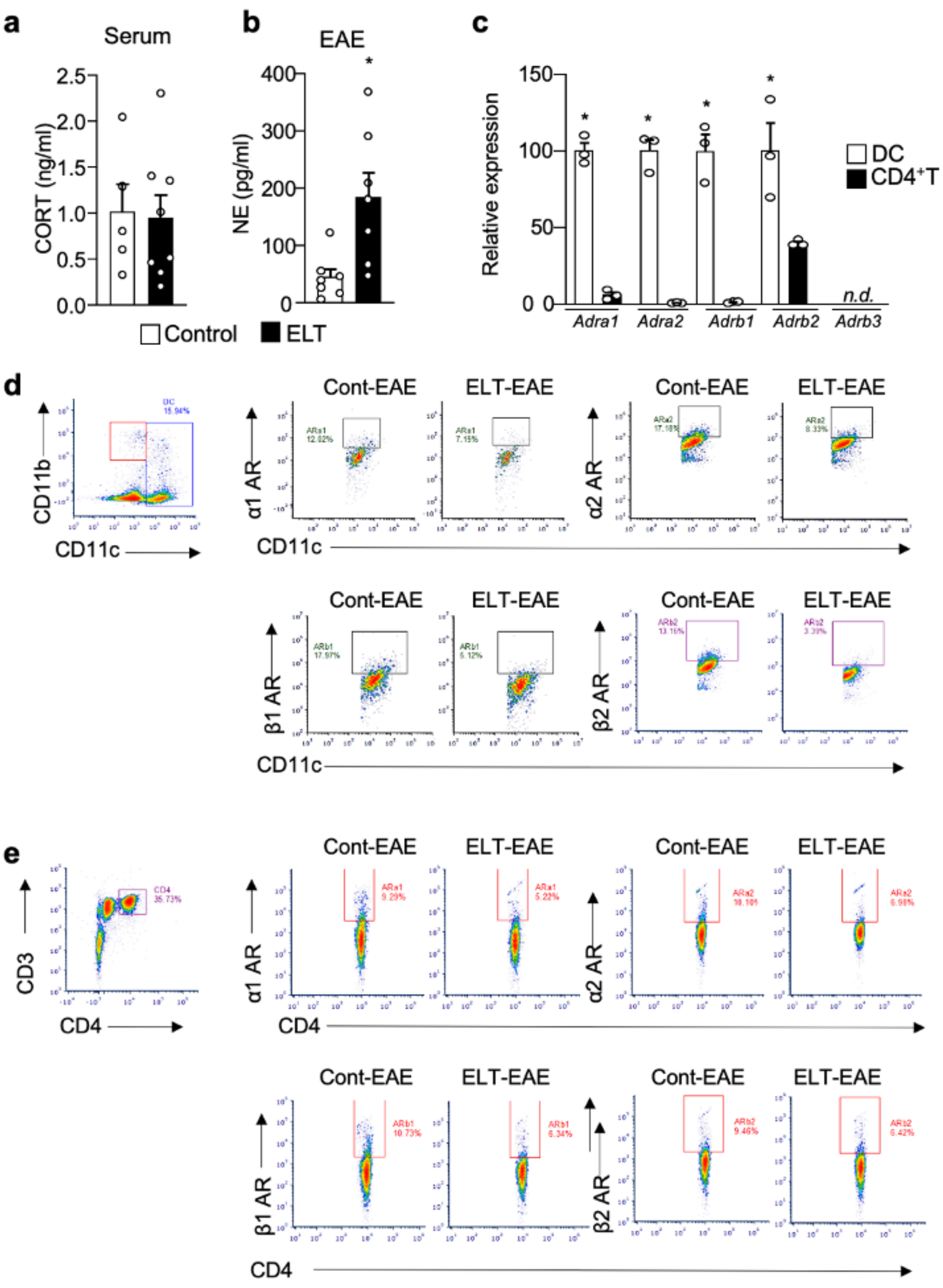

Supplementary Figure 6: Serum corticosterone levels and adrenergic-receptor mRNA expression on naïve dendritic cells and T cells.

(a) Corticosterone levels in serum in 4-week-old control and mice with ELT at 9 dpi (Cont; n=5, ELT; n=8).

(b) Norepinephrine levels in plasma in 10 dpi control EAE and ELT EAE mice (n=7 animals/group).

(c) Expression levels of adrenergic receptor subtype mRNA in DC and $CD4^{+}T$ isolated from lymph nodes of control mice (n=3 per group). Data were normalized by β-actin housekeeping genes.

(d, e) Representative gating strategy illustrating dendritic cell ($CD11c^{+}$) and $CD4^{+}T$ ($CD3^{+}$ $CD4^{+}$) expression of adrenergic receptors.

Data is represented as mean ± SEM. Student's t-test, $^{*}P < 0.05$. Exact p-values for asterisks: **(b)** 0.0109.

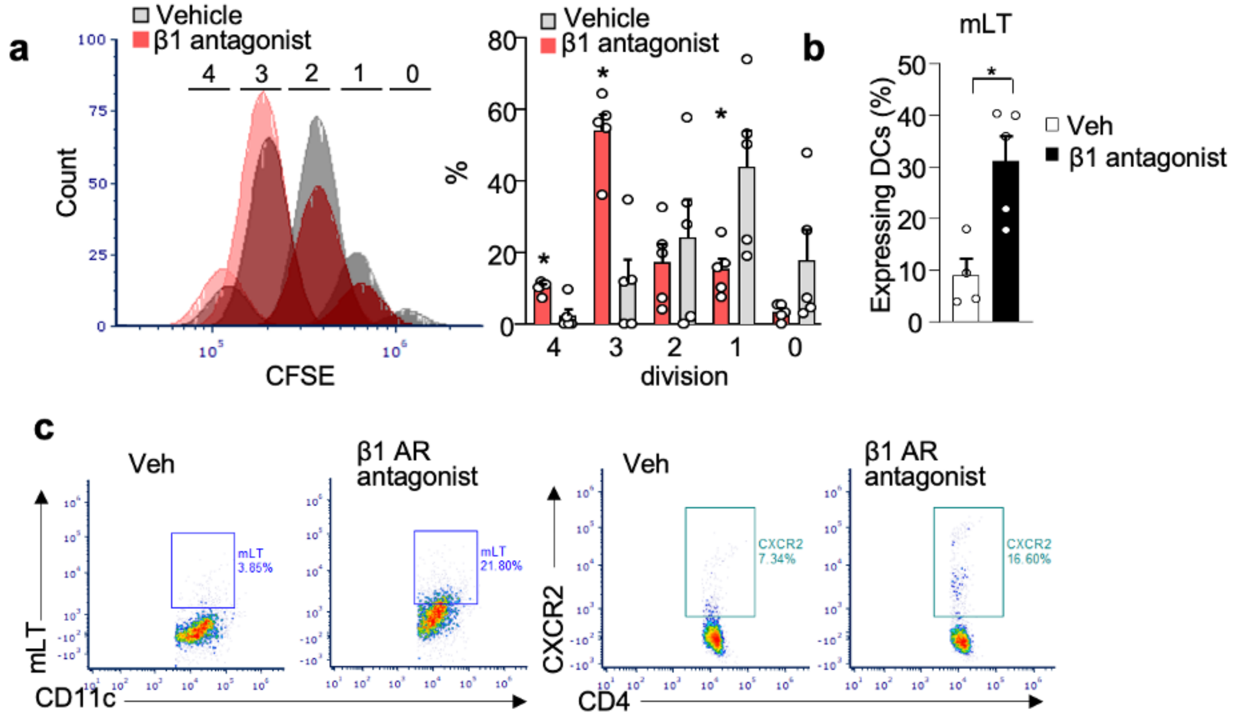

Supplementary Figure 7: β1-AR antagonist alters cell proliferation and DC mLT expression.
(a) Representative images of CD4$^+$T cells proliferation. CD4$^+$T cells (CD3$^+$CD4$^+$) were isolated from the lymph nodes of control EAE mice treated with and without β1-AR antagonist, labeled with CFSE proliferation kit, and co-culture with DC isolated from the lymph nodes of control EAE mice treated with and without β1-AR antagonist (n=5 animals/group).
(b) Percentage of mLT-expressing dendritic cells (DC: CD11c$^+$) at 10 dpi of EAE mice with and without β-AR antagonist (n=5 animals/group).
(c) A representative gating strategy illustrating dendritic cell (CD11c$^+$) expression of mLT and CD4$^+$T cell (CD3$^+$CD4$^+$) expression of CXCR2.
Data is represented as mean ± SEM. Student's t-test, *P < 0.05. Exact p-values for asterisks: (a) 0.041, 0.0007, 0.0267 (b) 0.0159.

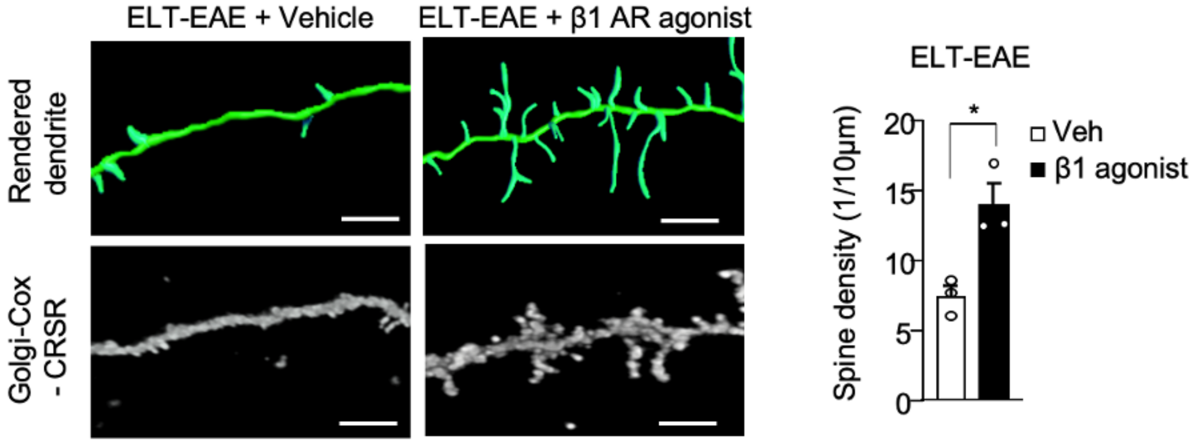

Supplementary Figure 8: β1-agonist significantly rescues dendritic spine loss effect.

CRSR imaging of Golgi-Cox stained dendrites in lumbar spinal cord ventral horn region with quantification of dendritic spine density at 30 dpi (n=3 animals/group). Data is represented as mean ± SEM. Scale bar represents 5μm. Student's t-test, **P* < 0.05. Exact p-values for asterisks: <0.001.

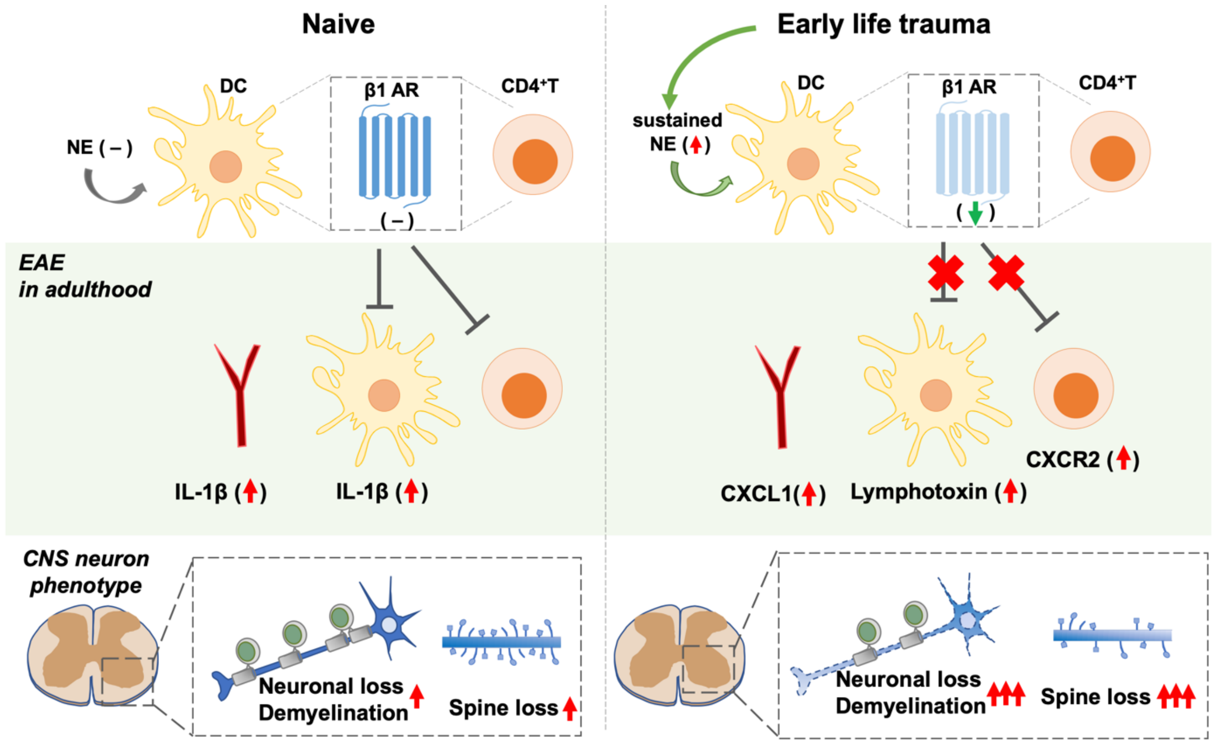

Supplementary Figure 9: Schematic depicting effect of ELT on EAE phenotype

Neonatal mice that were subjected to ELT sustained high circulating levels of norepinephrine (NE) and downregulation and adrenergic receptor expression in DC and T cell. ELT-mediated EAE presents with heightened lymphotoxin expression in DC and CXCR2 expression in T cell. ELT-mediated EAE presents with a more severe neuron damage phenotype in the spinal cord when compared with control EAE animals.

Supplementary table 1. List of reagents and antibodies

Reagents	Vendor	Catalog no.
MEVGWYRSPFSRVVHLYRNGK (100 mg)	United Peptides	MOG35-55
Complete Freund's adjuvant	Sigma	F5881-10X10ML
Heat-killed Mycobacteria	BD Difco	DF3114-33-8
Pertussis toxin	Fisher	NC9675592
FD Neurotechnologies INC FD Rapid Golgistain kit small	Fisher	NC0292960
Fluoro-Jade® C	Fisher	AG32530MG
Luxol Fast Blue MBSN, pure, ACROS Organics™	VWR	AC212170250
EasySep™ Mouse Biotin Positive Selection Kit	StemCell	18556
CXCR2 inhibitor	Tocris	SB225002
Interferon beta	Bayer	BETASERON®
Cirazoline hydrochloride, Tocris Bioscience	Fisher	08-881-0
Medetomidine hydrochloride, Tocris Bioscience	Fisher	51-601-0
Xamoterol hemifumarate, Tocris Bioscienc	Fisher	09-501-0
Procaterol hydrochloride, Tocris Bioscience	Fisher	11-021-0
Prazosin hydrochloride, Tocris Bioscience	Fisher	06-231-00
Selleck Chemical LLCSupplier Diversity Partner ATIPAMEZOLE 10MG	Fisher	50-136-4109
Metoprolol tartrate, Tocris Bioscience	Fisher	32-565-0
Selleck Chemical LLCSupplier Diversity Partner ICI-118551 HYDROCHLORIDE 5MG	Fisher	50-136-5671
Propranolol hydrochloride	Fisher	06-241-00
TRAF3 shRNA (m) Lentiviral Particles	SantaCruz	sc-36712-V
Control shRNA Lentiviral Particles-A	SantaCruz	sc-108080
LIFE TECHNOLOGIES CELLTRACE CFSE CELL PROLIFERAT	Fisher	50-591-407
CellTrace™ Violet Cell Proliferation Kit, for flow cytometry	Fisher	C34571
β-adrenergic receptor antagonist, propranolol		

Antibodies	Vendor	Catalog no.
PE/Cy7 anti-mouse CD19 Antibody	Biolegend	115520
Alexa Fluor® 700 anti-mouse CD3 Antibody	Biolegend	100216
Pacific Blue™ anti-mouse CD4 Antibody	Biolegend	100428
APC anti-mouse CD11b antibody	Biolegend	101212
PE anti-mouse CD8a antibody	Biolegend	100708
APC/Cyanine7 anti-mouse CD11c Antibody	Biolegend	117324
PE/Cy7 anti-mouse I-A/I-E Antibody	Biolegend	107630
FITC anti-mouse F4/80	Biolegend	123108
Ly-6G (Gr-1) Monoclonal Antibody (RB6-8C5), eFluor 450, eBioscience™	Fisher	48-5931-82
CD11b Monoclonal Antibody (M1/70), Alexa Fluor 700, eBioscience™	Fisher	56-0112-82
Pacific Blue™ anti-mouse Ly-6G Antibody	Biolegend	127612
PE anti-mouse Ly-6G Antibody	Biolegend	127608
FITC anti-mouse CD80 Antibody	Biolegend	104706
PE anti-mouse 4-1BB Ligand (CD137L) Antibody	Biolegend	107105
CD45 Monoclonal Antibody (30-F11), APC-eFluor 780, eBioscience™	Fisher	47-0451-82
Pacific Blue™ anti-mouse CD3ε Antibody	Biolegend	100334
CD4 Monoclonal Antibody (GK1.5), PE-Cyanine7, eBioscience™	Fisher	25-0041-82
APC anti-mouse CD3 antibody	Biolegend	100236
PE anti-mouse IL-17A Antibody	Biolegend	506904
Anti-mouse IFNg FITC	Biolegend	505806
PE/Cyanine7 anti-mouse/human CD11b Antibody	Biolegend	101216
PE anti-human CD11c Antibody	Biolegend	301606
FITC anti-mouse CD11c Antibody	Biolegend	117306
Goat Polyclonal Lymphotoxin-alpha/TNF-beta Antibody [Biotin]	Fisher	BAF749
Streptavidin-APC	Biolegend	405207
PE/Cyanine5 anti-mouse CD3ε Antibody	Biolegend	100309
Pacific Blue™ anti-mouse CD3ε Antibody	Biolegend	155612
FITC anti-mouse CD4 Antibody	Biolegend	100406
Alexa Fluor® 647 anti-mouse CD182 (CXCR2) Antibody	Biolegend	49306
Adrenergic, beta-1-, Receptor (ADRB1) (AA 197-222) antibody	Antibodies online.com	ABIN669351
Anti-ADRA2A antibody (Adrenergic, alpha-2A-, Receptor) (C-Term)	Antibodies online.com	ABIN1849124
Anti-beta 2 Adrenergic Receptor antibody (Adrenergic, beta-2-, Receptor, Surface)	Antibodies online.com	ABIN730153
Adrenergic Receptor, alpha 1d (ADRA1D) (C-Term) antibody	Antibodies online.com	ABIN4964908
IgG (H+L) Cross-Adsorbed Goat anti-Rabbit, Alexa Fluor® 488, Invitrogen	Fisher	A11008
Choline acetyltransferase ab (goat)	Fisher	PIPA14710
Goat Polyclonal AIF-1/Iba1 Antibody	Novus	NB100-1028
Donkey anti-Goat IgG (H+L) Secondary Antibody, Alexa Fluor 647, Invitrogen	Fisher	A21447
Chicken anti-Goat IgG (H+L) Cross-Adsorbed, Alexa Fluor 488, Polyclonal, Secondary Antibody, Invitrogen	Fisher	A21467

ELISA kits	Vendor	Catalog no.
Mouse GM-CSF DuoSet ELISA	Fisher	DY415
Mouse IFN-gamma DuoSet ELISA	Fisher	DY485
Mouse IL-2 DuoSet ELISA	Fisher	DY402
Mouse IL-17 DuoSet ELISA	Fisher	DY421
Mouse IL-1β ELISA MAX™ Standard	Biolegend	432602
R&D Systems™ Mouse CXCL1/KC DuoSet, R&D Systems™	Fisher	DY453
NA/NE ELISA	Fisher	50-148-9211
Abcam CAMP DIRECT IMMUNOASSAY KIT	Fisher	NC1070531

Supplementary Table 1: List of reagents and antibodies

List of primers

Primer name	Sequence 5' to 3'
Actb (b-actin)-Fw	TGT TAC CAA CTG GGA CGA CA
Actb (b-actin)-Rev	CTG GGT CAT CTT TTC ACG GT
Lta-F	TCA GAA GCA CTT GAC CCA TG
Lta-R	TCA AAG AGA AGC CAT GTC GG
Adra1d-F	GTG TCT TCG TCC TGT GCT G
Adra1d-R	GTA GAT GAG CGG GTT CAC AC
Adra2f F	CTC GCT GAA CCC TGT TAT CTA C
Adra2f R	TGC GTC TGA CCA TTG TCT G
Adrb1-F	ACT TCG GTA GAT GTG CTG TG
Adrb1-R	AAA CTC TGG TAG CGA AAG GG
Adrb2-F	CAT GGA AGG CTT TGT GAA CTG
Adrb2-R	GTC TGG TTA GTG TCC TGT CAA G
Adrb3-F	TGA TGG CTA TGA AGG TGC G
Adrb3-R	AAA ATC CCC AGA AGT CCT GC

Supplementary Table 2: List of primers
