# Supplementary material for: Early-life-trauma triggers interferon-β resistance and neurodegeneration in a multiple sclerosis model via downregulated β1-adrenergic signaling
Source: Nat Commun. 2021 Jan 4;12:105. doi: 10.1038/s41467-020-20302-0 (PMC7782805; doi:10.1038/s41467-020-20302-0)
Supplement: Supplementary file 3 — Reporting Summary [file 41467_2020_20302_MOESM3_ESM.pdf]

nature research

Corresponding author(s): NCOMMS-20-02170C

Last updated by author(s): Nov 6, 2020

Reporting Summary

Nature Research wishes to improve the reproducibility of the work that we publish. This form provides structure for consistency and transparency in reporting. For further information on Nature Research policies, see our Editorial Policies and the Editorial Policy Checklist.

Statistics

For all statistical analyses, confirm that the following items are present in the figure legend, table legend, main text, or Methods section.

n/a	Confirmed	
☐	☒	The exact sample size (n) for each experimental group/condition, given as a discrete number and unit of measurement
☐	☒	A statement on whether measurements were taken from distinct samples or whether the same sample was measured repeatedly
☐	☒	The statistical test(s) used AND whether they are one- or two-sided<br>*Only common tests should be described solely by name; describe more complex techniques in the Methods section.*
☒	☐	A description of all covariates tested
☒	☐	A description of any assumptions or corrections, such as tests of normality and adjustment for multiple comparisons
☐	☒	A full description of the statistical parameters including central tendency (e.g. means) or other basic estimates (e.g. regression coefficient) AND variation (e.g. standard deviation) or associated estimates of uncertainty (e.g. confidence intervals)
☐	☒	For null hypothesis testing, the test statistic (e.g. F, t, r) with confidence intervals, effect sizes, degrees of freedom and P value noted<br>*Give P values as exact values whenever suitable.*
☒	☐	For Bayesian analysis, information on the choice of priors and Markov chain Monte Carlo settings
☒	☐	For hierarchical and complex designs, identification of the appropriate level for tests and full reporting of outcomes
☒	☐	Estimates of effect sizes (e.g. Cohen's d, Pearson's r), indicating how they were calculated

Our web collection on statistics for biologists contains articles on many of the points above.

Software and code

Policy information about availability of computer code

Data collection	Fluorescent signals and Golgi-Cox stain were detected by using Nikon A1 confocal laser scanning fluorescence microscope. QPCR results were collected using QuantStudio™ 3 Real-Time PCR System. Fixed stained cell samples for flow cytometry were collected using Cytek Aurora. Western blots were visualized using FluorChem Systems, Bioteche.
Data analysis	Stereological image analyses were conducted using ImageJ. Spine density was analyzed using Imaris software and morpholibj plugin. The statistical analysis for all experiments was performed using GraphPad Prism.

For manuscripts utilizing custom algorithms or software that are central to the research but not yet described in published literature, software must be made available to editors and reviewers. We strongly encourage code deposition in a community repository (e.g. GitHub). See the Nature Research guidelines for submitting code & software for further information.

Data

Policy information about availability of data

All manuscripts must include a data availability statement. This statement should provide the following information, where applicable:

- Accession codes, unique identifiers, or web links for publicly available datasets
- A list of figures that have associated raw data
- A description of any restrictions on data availability

All data are presented in source data file. All relevant data are available from the corresponding author upon reasonable request. A reporting summary for this article is available as a Supplementary Information file.

Field-specific reporting

Please select the one below that is the best fit for your research. If you are not sure, read the appropriate sections before making your selection.

- [x] Life sciences
- [ ] Behavioural & social sciences
- [ ] Ecological, evolutionary & environmental sciences

For a reference copy of the document with all sections, see nature.com/documents/nr-reporting-summary-flat.pdf

Life sciences study design

All studies must disclose on these points even when the disclosure is negative.

Sample size	No statistical methods were used to predetermine sample sizes, but our sample sizes are similar to those generally employed in the field such as work by Shirley Yan et. al. 2003 published in Nature Medicine, Makoto Inoue et al. 2016 published in Nature Neuroscience, and Lei Zhou et. al. 2020 published in Nature Cellular and Molecular Immunology. Data distribution was assumed to be normal, but this was not formally tested.
Data exclusions	No data is excluded from study.
Replication	Most experiments were repeated for at least 1 time to ensure reproducibility. All replications were consistent.
Randomization	Animals were randomly selected in condition allocation process.
Blinding	Investigators were blinded to group allocation during experiment and analysis.

Reporting for specific materials, systems and methods

We require information from authors about some types of materials, experimental systems and methods used in many studies. Here, indicate whether each material, system or method listed is relevant to your study. If you are not sure if a list item applies to your research, read the appropriate section before selecting a response.

Materials & experimental systems

n/a	Involved in the study	
[ ]	[x]	Antibodies
[x]	[ ]	Eukaryotic cell lines
[x]	[ ]	Palaeontology and archaeology
[ ]	[x]	Animals and other organisms
[x]	[ ]	Human research participants
[x]	[ ]	Clinical data
[x]	[ ]	Dual use research of concern

Methods

n/a	Involved in the study	
[x]	[ ]	ChIP-seq
[ ]	[x]	Flow cytometry
[x]	[ ]	MRI-based neuroimaging

Antibodies

Antibodies used

Flow cytometry:
PE/Cy7 anti-mouse CD19 Antibody (Biolegend, 115520, 1:200), Alexa Fluor® 700 anti-mouse CD3 Antibody (Biolegend, 100216,1:200), Pacific Blue™ anti-mouse CD4 Antibody (Biolegend, 100428, 1:200), APC anti-mouse CD11b antibody (Biolegend, 101212, 1:200), PE anti-mouse CD8a antibody (Biolegend, 100708, 1:200), APC/Cyanine7 anti-mouse CD11c Antibody (Biolegend, 117324, 1:200), PE/Cy7 anti-mouse I-A/I-E Antibody (Biolegend, 107630, 1:200), FITC anti-mouse F4/80 (Biolegend, 123108, 1:200),Ly-6G (Gr-1) Monoclonal Antibody (RB6-8C5), eFluor 450, eBioscience™ (Biolegend, 48-5931-82, 1:200),CD11b Monoclonal Antibody (M1/70), Alexa Fluor 700, eBioscience™ (Biolegend, 56-0112-82, 1:200), Pacific Blue™ anti-mouse Ly-6G Antibody (Biolegend, 127612, 1:200), PE anti-mouse Ly-6G Antibody (Biolegend, 127608, 1:200), FITC anti-mouse CD80 Antibody (Biolegend, 104706, 1:200), PE anti-mouse 4-1BB Ligand (CD137L) Antibody (Biolegend, 107105, 1:200),CD45 Monoclonal Antibody (30-F11), APC-eFluor 780, eBioscience™ (eBioscience, 47-0451-82, 1:200), Pacific Blue™ anti-mouse CD3ε Antibody (Biolegend, 100334, 1:200),CD4 Monoclonal Antibody (GK1.5), PE-Cyanine7, eBioscience™ (eBioscience, 25-0041-82, 1:200), APC anti-mouse CD3 antibody (Biolegend, 100236, 1:200), PE anti-mouse IL-17A Antibody (Biolegend, 506904, 1:200), Anti-mouse IFNg FITC (Biolegend, 505806, 1:200), PE/Cyanine7 anti-mouse/human CD11b Antibody (Biolegend, 101216, 1:200), PE anti-human CD11c Antibody (Biolegend, 301606, 1:200), FITC anti-mouse CD11c Antibody (Biolegend, 117306, 1:200), Goat Polyclonal Lymphotoxin-alpha/TNF-beta Antibody [Biotin] (Biolegend, BAF749, 1:200, Streptavidin-APC (Biolegend, 405207, 1:200), PE/Cyanine5 anti-mouse CD3ε Antibody (Biolegend, 100309, 1:200), FITC anti-mouse CD4 Antibody (Biolegend, 100406, 1:200), Alexa Fluor® 647 anti-mouse CD182 (CXCR2) Antibody (Biolegend, 49306, 1:200), Adrenergic, beta-1-, Receptor (ADRB1) (AA 197-222) antibody (Antibodies online.com, ABIN669351, 1:200), Anti-ADRA2A antibody (Adrenergic, alpha-2A-, Receptor) (C-Term) (Antibodies online.com, ABIN1849124, 1:200), Anti-beta 2 Adrenergic Receptor antibody (Adrenergic, beta-2-, Receptor, Surface) (Antibodies online.com, ABIN730153, 1:200), Adrenergic Receptor, alpha 1d (ADRA1D) (C-Term) antibody (Antibodies online.com, ABIN4964908, 1:200), IgG (H+L) Cross-Adsorbed Goat anti-Rabbit, Alexa Fluor® 488, Invitrogen (Fisher, A11008, 1:500).

Western blots:
TRAF3 Rabbit anti-Gerbils, Human, Mouse, Rat, Polyclonal Antibody, Abnova™ (Fisher, 89-114-098, 1:1000). Rabbit anti-beta-actin (MyBioSource, MBS8507421, 1:1000).

Immunohistochemistry:
Choline acetyltransferase ab (goat) (Fisher, PIPA14710, 1:200), Goat Polyclonal AIF-1/Iba1 Antibody (Novus, NB100-1028, 1:300), Donkey anti-Goat IgG (H+L) Secondary Antibody, Alexa Fluor 647, Invitrogen (Fisher, A21447, 1:500), Chicken anti-Goat IgG (H+L) Cross-Adsorbed, Alexa Fluor 488, Polyclonal, Secondary Antibody, Invitrogen (Fisher, A21467, 1:500).

Validation

PE/Cy7 anti-mouse CD19 Antibody has been cited in 38 publications. Alexa Fluor® 700 anti-mouse CD3 Antibody has been cited by 39 publications. Pacific Blue™ anti-mouse CD4 Antibody has been cited by 34 publications. APC anti-mouse CD11b antibody has been cited by 135 publications. PE anti-mouse CD8a antibody has been cited by 85 publications. APC/Cyanine7 anti-mouse CD11c Antibody has been cited by 52 publications. PE/Cy7 anti-mouse I-A/I-E Antibody has been cited by 21 publications. FITC anti-mouse F4/80 has been cited by 99 publications. Ly-6G (Gr-1) Monoclonal Antibody (RB6-8C5), eFluor 450, eBioscience™ has been cited by 108 publications and has 40 published figures. CD11b Monoclonal Antibody (M1/70), Alexa Fluor 700, eBioscience™ has been cited by 185 publications and has 40 published figures. Pacific Blue™ anti-mouse Ly-6G Antibody has been cited by 33 publications. PE anti-mouse Ly-6G Antibody has been cited by 91 publications. FITC anti-mouse CD80 Antibody has been cited by 24 publications. PE anti-mouse 4-1BB Ligand (CD137L) Antibody has been cited by 4 publications. CD45 Monoclonal Antibody (30-F11), APC-eFluor 780, eBioscience™ has been cited by 59 publications. Pacific Blue™ anti-mouse CD3ε Antibody has been cited by 8 publications. CD4 Monoclonal Antibody (GK1.5), PE-Cyanine7, eBioscience™ has been cited by 100 publications. APC anti-mouse CD3 antibody has been cited by 42 publications. PE anti-mouse IL-17A Antibody has been cited by 61 publications. Anti-mouse IFNg FITC has been cited by 53 publications. PE/Cyanine7 anti-mouse/human CD11b Antibody has been cited by 113 publications. PE anti-human CD11c Antibody has been cited by 6 publications. FITC anti-mouse CD11c Antibody has been cited by 47 publications. Goat Polyclonal Lymphotoxin-alpha/TNF-beta Antibody [Biotin] has no review thus far. Streptavidin-APC has been cited by 39 publications. PE/Cyanine5 anti-mouse CD3ε Antibody has been cited by 17 publications. Pacific Blue™ anti-mouse CD3ε Antibody has not yet been cited. FITC anti-mouse CD4 Antibody has been cited by 70 publications. Alexa Fluor® 647 anti-mouse CD182 (CXCR2) Antibody has been cited by 2 publications. Adrenergic, beta-1-, Receptor (ADRB1) (AA 197-222) antibody has been cited by 2 publications. Anti-ADRA2A antibody (Adrenergic, alpha-2A-, Receptor) (C-Term) has not yet been reviewed. Anti-beta 2 Adrenergic Receptor antibody (Adrenergic, beta-2-, Receptor, Surface) has not yet been reviewed. Adrenergic Receptor, alpha 1d (ADRA1D) (C-Term) antibody has not been reviewed. IgG (H+L) Cross-Adsorbed Goat anti-Rabbit, Alexa Fluor® 488, Invitrogen has been cited by 189 publications. Choline acetyltransferase ab (goat) has been cited by 23 publications. Goat Polyclonal AIF-1/Iba1 Antibody has been cited by 154 publications. Donkey anti-Goat IgG (H+L) Secondary Antibody, Alexa Fluor 647, Invitrogen has been cited by 60 publications. Chicken anti-Goat IgG (H+L) Cross-Adsorbed, Alexa Fluor 488, Polyclonal, Secondary Antibody, Invitrogen has been cited by 33 publications. TRAF3 Rabbit anti-Gerbils, Human, Mouse, Rat, Polyclonal Antibody, Abnova™ has been been cited by at least 1 publication. Rabbit anti-beta-actin has been been cited by at least 1 publication.

Animals and other organisms

Policy information about studies involving animals; ARRIVE guidelines recommended for reporting animal research

Laboratory animals

Healthy C57BL/6J mice aged 6–8 weeks were used in this study. In most experiments, male mice were used. In some experiments, female mice were also used. C57BL/6J mice and TCR2D2 mice (Jackson Laboratory, #6912) were purchased from Jackson Laboratories. Adrb1 fl/fl micewere a gift from Dr. Zigman (The University of Texas Southwestern Medical Center) under material transfer agreement (MTA)83. DC-specific and T cell-specificAdrb1-/-mice were generated by crossing Adrb1 fl/fl mice with Itgax-Cre (Jackson Laboratory 008068) or Lckicre mice (Jackson Laboratory 012837) mice, respectively. Asc-/-mice were a gift Genentech under MTA. All mice were kept in group housing (3–5 mice per cage) in a specific pathogen-free facility with a 12-h light/dark cycle and temperature control at 20-22ºCat the Veterinary Medicine Basic Sciences Building at the University of Illinois at Urbana-Champaign. Breeders (one male with one female) were housed in solid-bottom caging with standard bedding. Rodent diet (Teklad) and water were provided ad libtum. Mouse litter size and sex ratio varies between breeders and pregnancies. Male mice were used for experiments unless otherwise mentioned. The study was approved by the University of Illinois Institutional Animal Care and Use Committee (protocol number 19171).

Wild animals

No wild animals were used.

Field-collected samples

There are no field collected samples.

Ethics oversight

The study was approved by the University of Illinois Institutional Animal Care and Use Committee.

Note that full information on the approval of the study protocol must also be provided in the manuscript.

Flow Cytometry

Plots

Confirm that:

- [x] The axis labels state the marker and fluorochrome used (e.g. CD4-FITC).
- [x] The axis scales are clearly visible. Include numbers along axes only for bottom left plot of group (a 'group' is an analysis of identical markers).
- [x] All plots are contour plots with outliers or pseudocolor plots.
- [x] A numerical value for number of cells or percentage (with statistics) is provided.

Methodology

Sample preparation

For western blot:
Tissues were excised from mice and cells from culture systems were kept frozen at -80ºC. Tissues were homogenized by a homogenizer. To normalize TRAF3 signals by beta-actin in same blotting membrane, membrane was cut, and was stained with specific antibodies.

For ELISA:
Blood samples were taken at approximately 9 am from all mice to ensure consistency in sample quality. Serum or plasma were isolated and stored at 80ºC until ELISA analysis.

For flow cytometry:
The spleen, lymph nodes (axillary and inguinal), brain, and spinal cord were removed, then minced with a sterile razor blade. Minced spleen and lymph nodes were passed through a mesh filter. Brain and spinal cord were digested in collagenase for 30 min at 37 °C, and then passed through a 70-µm filter to remove debris. CNS-infiltrating lymphocytes were isolated by percoll gradient centrifugation. After washing, the cells were suspended in ice-cold staining buffer (sterile PBS containing 2% FBS). Cells were blocked with anti-CD16/32 on ice for 10 min. After blocking, 1 × 10^6 cells were suspended in 0.1 mL of ice-cold staining antibody buffer and stained on ice in the dark for 20 min. After washing twice in staining buffer, the samples were kept in 4% PFA until cells are acquired on a Cytek Aurora flow cytometer.

For DNA methylation assay:
DCs (CD11c+) were isolated from inguinal and axillary lymph nodes by beads positive selection (EasySepMouse Biotin Positive Selection kit) with Biotinylated CD11c antibody from spleen of control-EAE and ELT-EAE mice at EAE at 9-dpi. Genomic DNA was obtained with the GenEluteTMMammalian Genomic DNA Miniprep kit (Sigma).

For bone marrow-derived cells and cellular differentiation:
Bone marrow was isolated from naïve C57BL/6Jmice. Isolated cells were treated with recombinant GM-CSF (200 ng/ml) for 6 days to generate bone marrow-derived DCs (BMDC). For qPCR experiment, BMDC cells were treated with AR agonists (10 µg/ml) for 18 hours at 37°C then treated with Mtb (100g/ml) or lipopolysaccharides (1 µg/ml) for 3 hours at 37°C. As control, BMDC were treated with vehicle (PBS) instead of AR agonists, and vehicle instead of Mtb or LPS.

For immunohistochemistry:
Spinal cords were harvested from PBS-perfused and 4% paraformaldehyde-fixed mice at 30 dpi. Spinal cords were post-fixed in 4% paraformaldehyde overnight and then cryoprotected by immersion in 30% sucrose solution for 24 h. Samples were frozen in OCT compound and stored at -80ºC until cryostat sectioning. Transverse sections (30-µm) of spinal cords were mounted on poly-L-lysine-coated glass slides.

For Golgi-Cox staining:
Mice were deeply anesthetized with isoflurane and perfused intra-cardially with Phosphate buffered saline (PBS) (15 ml) followed by 4% paraformaldehyde (pH 7.4) (25 ml). Spinal cord was removed and transferred into solutions designation by the FD Rapid Golgi-Stain Kit (FD Neuro-Technologies, INC).

Instrument

For western blot:
FluorChem Systems, Bioteche, Trans-Blot® Turbo, Bio-Rad

For flow cytometry:
Cytek Aurora, Aurora

For DNA methylation assay:
Electrophoresis Horz W/BEP, Fisher Scientific; T100™ Thermal Cycler, Bio-Rad

For immunohistochemistry:
Confocal laser scanning fluorescence microscope, Nikon A1

Software

For western blot:
ImageJ

For immunohistochemistry:
ImageJ, Fiji

For Confocal reflection microscopy:
Imaris

For flow cytometry:
FCS express version 6

	For statistics:<br>Graphpad prism version 8.
Cell population abundance	The cells were fixed by 4% paraformaldehyde for no more than 72h prior to acquisiton by flow cytometer.
Gating strategy	The gating strategy (FSC-A vs SSC-A) was used to exclude cell debris and aggregates. The same gating strategy were applied to both control and experiment conditions.

☒ Tick this box to confirm that a figure exemplifying the gating strategy is provided in the Supplementary Information.
